# Nanobioengineered Sensing Technologies Based on Cellulose Matrices for Detection of Small Molecules, Macromolecules, and Cells

**DOI:** 10.3390/bios11060168

**Published:** 2021-05-24

**Authors:** Divya, Supratim Mahapatra, Vinish Ranjan Srivastava, Pranjal Chandra

**Affiliations:** Laboratory of Bio-Physio Sensors and Nanobioengineering, School of Biochemical Engineering, Indian Institute of Technology (BHU) Varanasi, Varanasi 221005, Uttar Pradesh, India; divya.rs.bce20@itbhu.ac.in (D.); supratimmahapatra.rs.bce20@itbhu.ac.in (S.M.); vinishrsrivastava.bce20@iitbhu.ac.in (V.R.S.)

**Keywords:** nanobioengineering, cellulose, matrix design, biosensors, cytosensing, human health

## Abstract

Recent advancement has been accomplished in the field of biosensors through the modification of cellulose as a nano-engineered matrix material. To date, various techniques have been reported to develop cellulose-based matrices for fabricating different types of biosensors. Trends of involving cellulosic materials in paper-based multiplexing devices and microfluidic analytical technologies have increased because of their disposable, portable, biodegradable properties and cost-effectiveness. Cellulose also has potential in the development of cytosensors because of its various unique properties including biocompatibility. Such cellulose-based sensing devices are also being commercialized for various biomedical diagnostics in recent years and have also been considered as a method of choice in clinical laboratories and personalized diagnosis. In this paper, we have discussed the engineering aspects of cellulose-based sensors that have been reported where such matrices have been used to develop various analytical modules for the detection of small molecules, metal ions, macromolecules, and cells present in a diverse range of samples. Additionally, the developed cellulose-based biosensors and related analytical devices have been comprehensively described in tables with details of the sensing molecule, readout system, sensor configuration, response time, real sample, and their analytical performances.

## 1. Introduction

Cellulose is a biopolymer of β-1,4 D-glucose units, a widely used biomaterial that exhibits unique properties and is used in various industries including textiles, electronics, biomedical, etc. [1]. Cellulose is the most commonly occurring biomaterial on earth [2], obtained mainly from different plant species [3] and some form of bacteria [4], i.e., *Acetobacterxylinum* [5]. Glucose molecules are bonded with van der Waal forces and hydrogen bonding to form a parallel stacking of cellulose microfibrils into crystalline cellulose [6]. Due to its structural form, cellulose exhibits unique properties such as high young’s modulus, biocompatibility, biodegradability, high mechanical strength, transparency, and thermal stability, which allows high chemical modifications [7,8,9]. As well as appealing piezoelectricity, mechanical performance, dialectricity, and convertibility [10] have also been recorded. Cellulose has its application in a broad range of fields, such as in the pharmaceutical industry for coating of tablets, pellets, beads, granules [11], and in various other industries [12]. In addition to this, cellulose has also been widely used in the immobilization of different molecules, in wound healing, tissue engineering [13,14,15], and also has tremendous applications in flexible printed bioelectronics [16]. In recent times, further modification of cellulose with the nanostructures leads to its application as a matrix material for biosensor fabrication, which is generally called “nanocellulose”. Nanocellulose is a sustainable, renewable, and eco-friendly nanomaterial with remarkable properties such as high strength, low density, high specific surface area, high aspect ratio, etc. [17]. It is also worth mentioning that these properties are tunable, and they majorly rely on the chemical modification steps and type of nanomaterials integrated with cellulose matrix. These nano-cellulosic systems can be constructed using various methods such as chemical activation, grafting, coating, impregnation, covalent binding, cryocrushing, micro-fluidization, high-intensity ultrasonication and various other wet chemistry procedures [18]. They can be cellulose nanofibers (CNF), cellulose nanocrystal (CNC), or bacterial nanocellulose [19]. The remarkable properties of nanocellulose make the polymer of cellulose one of the most fascinating and advanced materials with implementation in various fields [20]. Cellulosic material possesses the complete degradation and biocompatible property due to which it is also called “green cellulose” [21] and is used for the fabrication of disposable and degradable sensors. The hydrogen bonding present in the core structure of cellulose provides both mechanical strength and flexibility at the same time; this unique property helps in the fabrication of a flexible matrix for sensors [22,23,24]. Due to such multidimensional properties and cost-effectiveness, cellulose has been considered as one of the most fascinating and widely used materials for the development of biosensors. For improving the sensitivity of the cellulose surface, novel modifications can be performed using conducting materials such as gold nanoparticles (AuNPs), carbon nanotubes (CNTs), gold-nanorods, graphene oxide (GO), and other nanocomposites [9,25].

**Figure 1:**
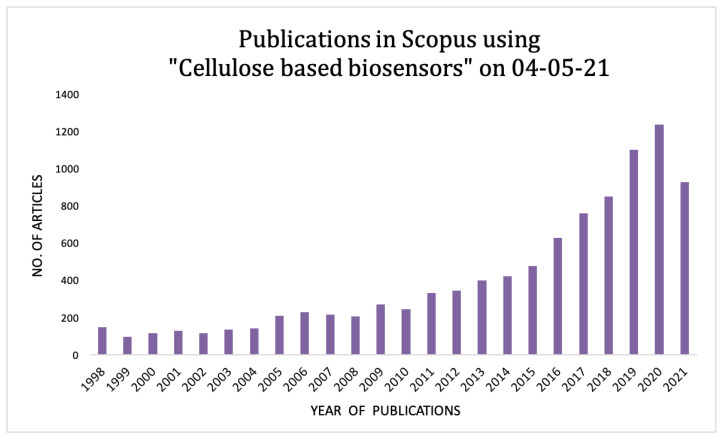
The number of articles published in consequent years in the online database “Scopus” using the keyword “cellulose-based biosensors”.

In this review, we have critically evaluated the cellulose-based sensors that have been fabricated through distinct modification techniques and their varying applications for analyzing diverse sample types including small molecules, ions, macromolecules, and cells. While the studies on cellulose-based biosensors continue, major advances have been achieved in this field. Thus, we have performed a scientific survey for the research documentation concerning cellulose-based sensors through the online database “Scopus”, the result of which is quite a convincing indicator that cellulose-based biosensors have gained a major focus in recent times considering their multipotent abilities. According to this survey, it was found that more than 7000 research articles have been published in the last decade, following an exponential increase (Figure 1). In the following sections, we have listed the introductory concepts of cellulose biosensors followed by their nanobioengineering design aspects for the detection of diverse molecules in various real sample matrices.

## 2. Cellulose: Structure and Biosensing Design Aspects

A biosensor is defined as an analytical device that detects the biological molecule through electrical, thermal, or optical signals [26,27]. It consists of four components—the bio-recognition element, transducer, amplifier, and detector. Biorecognition elements are composed of enzymes, cells, DNA, proteins, tissue, organelles, antibodies, aptamers, etc. [26,28]. The analyte, when starting to sinter, acts with a biorecognition element to produce a signal that is converted to readable form by the transducer [29]. Depending upon the types of transducer, biosensors could be categorized commonly into different kinds such as optical, electrochemical, electromechanical, calorimetric, acoustic biosensors, etc. Optical biosensors detect the change in optical properties such as phase change, polarization, and change in frequency of light. Mechanical biosensors detect the variation in mechanical properties such as mass, force, motion, etc. Electrochemical biosensors analyze the change in chemical stimuli to readable electrical signals [26,30,31]. Colorimetric biosensors detect the change in color through naked eyes or by using simple optical detector [32]. To date, several cellulose-based sensors have been developed based on the source of cellulose and various modifications were performed according to the required function. Interestingly, the cellulose and its composite have been successfully used as a component of various transducing surfaces for the development of these sensors. Cellulose and its derivatives are designed to introduce the desired characteristics through chemical functionalization and also by changing the inherited inter and intra hydrogen-bonding pattern. Various nanomaterials are also explored for enzyme-like properties to enhance the electrocatalytical activity in the designing of biosensors.

In the structure of cellulose, each glucose unit consists of three hydroxyl groups at which chemical modifications are performed. These modifications can be majorly performed on the surface without disturbing the main chain, which results in the formation of the required cellulosic matrix [33]. The immobilization of cellulose with AuNPs [34], CNTs [35], reduced GO [36], quantum dots (QDs) [37], and other nanomaterials help in the fabrication of biosensors with enhanced properties in a cost-effective manner [38,39]. CNTs and rGO provide enhanced electrical conductivity and high active surface area that helps in large number of bio-receptor immobilization. AuNPs helps to establish a plasmonic field, color change, fluorescence quenching, and also assist in enhancing the surface area as well as electron transfer of the sensing surface [40,41,42]; QDs have unique luminescence and electronic properties [43], so they are also used in cellulose-based sensing platforms. Bacterial cellulose (BC) is an eco-friendly, natural three-dimensional nanostructure, and a low-cost molecule derived from bacteria, which is also used as a matrix material in biosensor development [44]. Biosensors based on BC show various advantages over conventional cellulose-based sensors because of their immense surface area, higher crystallinity, good biocompatibility, and great mechanical strength [45]. Cellulose-based biosensors are widely accepted and used for the detection of various sorts of molecules such as small molecules, macromolecules, metals, and cells [9], etc. CNFs are used for microfluidic channel preparation and enzyme derivatization in the microfluidic-sensor matrices for effective biomedical diagnosis [46,47]. These microfluidic sensors are inexpensive, disposable, lightweight, and a rapid method of diagnostics that supports rheological modification and exhibit mechanical strength and advantageous dimensional stability [48,49]. In addition to these, cellulose in another form, such as paper, has also been explored, for example, Whatman filter paper [50,51], glossy papers [52], paper towels [53], etc. Glossy paper can be used in a paper-based flexible sensing device, as it is composed of cellulose fibers blended with inorganic compounds. The main advantage of using glossy paper is that it is easy to engineer its surface properties.

## 3. Biosensors for the Detection of Small Molecules and Metals

**Figure 2:**
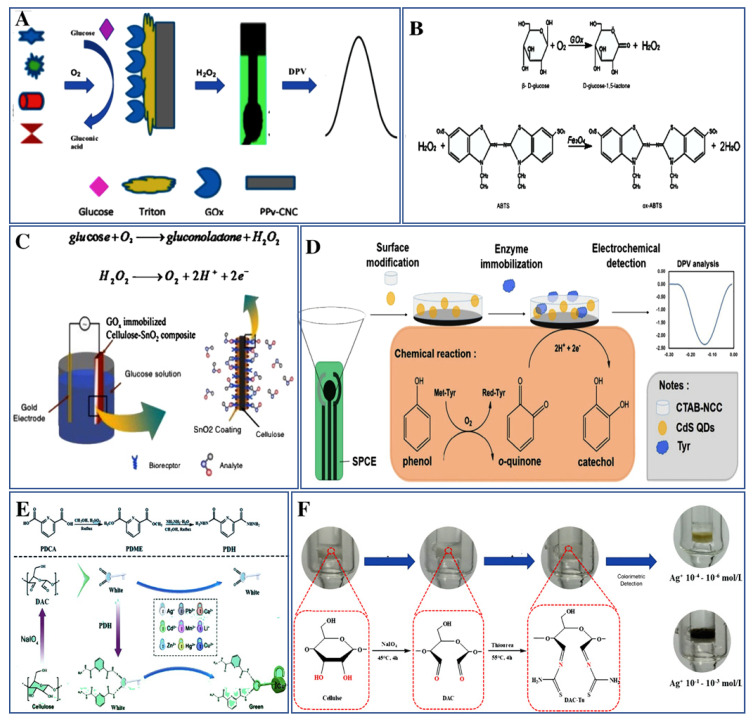
Biosensors for detection of small-molecule and metal ion. (**A**) Pictorial representation of poly pyrrole-cellulose nanocrystal (PPy-CNC)-based biosensor for the analysis of glucose and response recorded with a DPV (reproduced with permission from [68]). (**B**) Representation of the reaction mechanism of cellulose nanocrystal/magnetite glucose biosensor (Reproduced with permission from [55]). (**C**) Representation of cellulose–SnO_2_ nanocomposite-based biosensor for the detection of glucose molecule (reproduced with permission from [54]). (**D**) Pictorial representation of the development of CTAB-NCC/MPA-QDs/Tyr sensor through immobilization of CTAB-NCC/QDs on SPCE (screen-printed carbon electrode) and further with Tyr enzyme for the detection of phenol and response evaluated using DPV (reproduced with permission from [58]). (**E**) Fabrication scheme of DAC-PDH-based colorimetric sensor through chemical alteration and discriminatory identification of Cu^2+^ by DAC-PDH within 30 s (reproduced with permission from [69]). (**F**) Preparation of DAC-Tu selective colorimetric sensor and the identification of Ag^+^ from aqueous solutions according to color change based on the varying concentrations of Ag+ (reproduced with permission from [70]).

Small molecules are the low molecular weight organic molecules, normally less than 900 Daltons. They perform various types of biological functions such as a signaling molecule, drugs, effector, and even sometimes changes the function of the target. Due to their size, they readily diffuse across the membranes. Due to their wide range of functions, it is required to monitor the small molecules involved in maintaining the biological functions. In this section, cellulose-based biosensors that are developed for the determination of numerous small molecules have been discussed. One of the most important small molecules is glucose, which has a tremendous role in clinical laboratories and other industries. Glucose is a monosaccharide ubiquitously present in humans and acts as a fuel of human body, and when it is present in the blood, is called blood sugar [54]. To detect glucose, various cellulose-based sensors have been developed in recent years. A blood glucose biosensor for simple and affordable monitoring has been developed recently for its direct detection in sweat and saliva samples [55]. In this case, sulfated (S-CNC) and non-sulfated (N-CNC) cellulose nanocrystal/magnetite film was used to determine the instant color change in the presence of glucose and 2,2′-azino-bis (3-ethylbenzothiazoline-6-sulphonic acid) (ABTS). The authors used the peroxidase-like characteristics of (N-CNC)-Fe_3_O_4_ and (S-CNC)-Fe_3_O_4_ nanoparticles to determine glucose, using glucose oxidase (GOx) and H_2_O_2_ (Figure 2B). Here, ABTS was used as a substrate for the peroxidase. Both sulfated and non-sulfated types of biosensors detect the concentration of glucose as low as 5 mM showing high sensitivity and concentration compared to the glucose level present in biological fluids. Even sulfated nanoparticles showed 1.5 to 2 times more reactivity than that of non-sulfated systems. An ampero metric glucose biosensor has been developed by Lawrence et al., 2014, through immobilization of GOx, extracted from *Aspergillus niger*, in a paper disk matrix (diameter 1 cm) positioned on the surface of a screen-printed carbon electrode (SPCE) [21]. For glucose oxidation, ferrocene monocarboxylic acid was used as an intermediate molecule. The developed paper-based biosensor was used for detecting the glucose at an extremely low operating volume of 5 μL and concentration range between 1 to 5 mM. The limit of detection (LOD) of the developed sensor was 180 μM (*n* = 5 at 90% confidence level). In addition to these, other glucose biosensors are mentioned in Table 1 with information related to the sensor’s design, readout system, and other analytical details.

In addition to glucose, other molecules are also detected using a cellulose-based sensor such as phenol, which is a major component of various industries and industrial waste that leads to its deteriorating effects on the environment. Phenol is one such toxic pollutant present in wastewater, released from industrial products, and causes harmful effects to aquatic life, plants, and also humans, sometimes leading to organ damage [56,57]. An electrochemical cellulose-based biosensor has been developed for the sensing of phenol by Manan et al., 2019 [58]. The biosensor was fabricated through hybridization of cetyltrimethylammonium bromide (CTAB) on nano-crystalline cellulose (NCC) and further hybridization of CTAB-NCC to QDs capped with 3-mercaptopropionic acid (MPA). The developed nanocomposite material was immobilized with tyrosinase enzyme (Tyr) to form CTAB-NCC/MPA-QDs/Tyr for the determination of phenol (Figure 2D). The developed biosensor shows the dynamic range from 5 to 40 μM (0.47 to 3.76 mg/L) and the observed LOD of the sensor was 82 nM (7.7 μg/L). It shows high sensitivity up to 0.078 μA/μM and can be a potential biosensor for detection of phenol in surrounding samples. Apart from these, cellulosic matrix is also being used in the sensing of amines, which are important materials employed in the food industry, biological processes, and are harmful to the atmosphere and public health [59].

A cellulose-based fluorescent biosensor has been developed for the sensing of various amines by Nawaz et al., 2020. The biosensor was manufactured by using the phenanthroline (Phen) as a color-imparting molecule and 4,4′-methylene diphenyl diisocyanate (MDI) immobilized on cellulose acetate (CA) to form a Phen-MDI-CA sensor for the visualization of amines [60]. Different colors of the fluorescent molecule were observed for different amines on Phen-MDI-CA paper by using UV-visible light. For example, the blue color fluorescence of Phen-MDI-CA modified to light blue when triethylamine (TEA) was used as a substrate, to green for diethyl amine (DEA), and to cyan for methylamine. The observed LOD of the manufactured biosensor was found to be 900 nmol, 990 nmol, 1700 nmol for TEA, EDA, methylamine, respectively. Not only these molecules but cellulosic matrices has been also applied for the sensing of pesticides such as fluazinam, which is a low-toxic fungicide that poses a threat to the environment and food safety [61]. Wang et al., 2020 prepared a paper-based biosensor for sensing and digital analysis of fluazinam. The biosensor was fabricated by crosslinking the disulfide MoS_2_ QDs into cellulose membranes. The developed biosensor shows the detection range between 10 to 800 μM. The LOD observed for the fluazinam sensor was 2.26 μM [62]. Apart from fluazinam, cellulose-based sensors were explored for the sensing of other toxins. A label-free electrochemical biosensor was developed for the quantification of aflatoxin B1 (AFB1) in wheat samples by Huang et al., 2020. The author used CNFs derived from BC and coupled them with AuNPs. It produced a unique 3D porous structure that helps in the speedy diffusion of electrolytes, providing a greater electrochemical working are a that helps in obtaining higher current magnitude in a typical differential pulse voltammogram (DPV). The developed sensor shows the detection of AFB1 in a broad concentration range from 50 to 25 × 10^3^ pg mL^−1^ having a determination coefficient of 0.995. The estimated LOD for the immunosensor was observed to be 27 pg/mL [63]. The author also checked the reproducibility, stability, and selectivity of the biosensor and it was found to be suitable. As mentioned earlier, cellulose in its other forms, such as paper, which can be derived from wood, grasses, rags, and other sources, has also been widely used in sensing devices.

The cellulosic paper provides unique geometry and hydrophilic nature which are explored to develop paper-based analytical devices [64,65]. Phenylalanine (Phe) is an essential amino acid and failure of its metabolism causes phenylketonuria, which is due to the deficiency of phenylalanine hydroxylase enzyme, which converts Phe into tyrosine, due to which Phe is deposited in the human body and causes brain injury and neurocognitive dysfunction [66]. Sun et al., 2021, manufactured a paper-based biosensor for the identification of Phe using phenylalanine ammonia lyase hybrid nanoflowers [67]. The developed biosensor was capable of sensing Phe concentration in urine samples with detection range of 60 to 2400 μM within 10 min. In addition to these examples, sensors have also been developed for the detection of other small molecules, which has been comprehensively described in Table 1.

Cellulose-based biosensors are not simply used for the sensing of small molecules as discussed above; they are also used for the screening and detection of heavy metals. Heavy metals are detrimental to living beings beyond a particular concentration as they are not biodegradable and can accumulate in the body [71]. Cellulose-based biosensors have been designed for the sensing of various metal ions as well. A few examples that are specific and sensitive to a particular ion even in the presence of interfering molecules are discussed here. Copper ion (Cu^2+^) plays a significant role in different physiological processes, excess of which causes liver and kidney damage, and which is one of the frequently occurring ions causing the contamination of drinking water. A cellulose-based biosensor has been developed for the unaided detection of Cu^2+^ ions present in biological fluids by Wang et al., 2019 [69]. The biosensor was fabricated without the requirement of the probe using natural cellulose through a facile one-pot process, and structural characterization was carried out by Fourier transform infrared spectroscopy (FTIR). The sensitivity of the biosensor was detected in serum, urine, and water through the UV–vis absorption intensity and it shows the color change within 10 s. The LOD was concluded to be 1.9309, 1.9154 and 1.185 ppm in urine, serum, and tap water, respectively. The developed biosensor was established to be very effective in sensing Cu^2+^ ions in the biological fluids and showed rapid response time, high sensitivity, and selectivity (Figure 1E). Apart from Cu^2+^, other cellulose-based sensing systems have been developed for the other metal ion such as silver (Ag^+^), which is present in water and its high concentration causes adverse effects in the environment and on public health [72]. A cellulose-based colorimetric sensor has been fabricated for the detection of Ag^+^ in an aqueous solution by Wang et al., 2020 [70]. The biosensor was fabricated by embed ding thiourea (Tu) onto the surface of Eucalyptus cellulose, which is modified to dialdehyde cellulose (DAC) through a chemical grafting method. The visual detection method depends upon the chelation of Ag^+^ with N and S atoms of DAC-Tu to form N-Ag, S-Ag, and Ag_2_S and it shows different colors based on different concentrations of Ag^+^ (Figure 2F). The coloring mechanism between Ag^+^ and DAC-Tu in aqueous solution was characterized by nuclear magnetic resonance, transmission electron microscopy (TEM), and X-ray photoelectron spectroscopy (XPS) analysis. The signal response of the sensor reduces with an increase in the concentration of Ag^+^ and the LOD was found to be 10^−6^ mol/L within 10 min. Apart from these ions, cellulose-based sensing devices are also fabricated for the identification of zinc ion (Zn^2+^), which is found abundantly in the environment due to industrial processes and causes harm to human health. Such a cellulose-based optical biosensor was designed for the sensing of Zn^2+^ by Daniyal et al., 2019 [73]. The surface plasmon resonance (SPR)-based optical sensor was fabricated through enhancement of thin gold film with the help of NCC. The SPR signal was examined both with the unmodified and modified thin film of gold for determining the sensing potential. The Zn^2+^ interacts with the negative charge present on the modified-gold film and such an interaction has been investigated by XPS. The XPS scans into the region of 0 to 1400 eV and the recorded data were equipped with the Gaussian–Lorentzian curve program. The developed biosensor possessed a wide dynamic range with the LOD of 0.01 ppm and illustrates the sensitivity of about 1.892 ppm^−1^.

Interestingly, not only these ions but cellulose-based sensors have also been fabricated for the identification of mercury (Hg^2+^), which is a major threat to human health due to its toxicity. Timely detection is necessary to decrease the level of Hg^2+^ in industrial waste. Zheng et al., 2021, have prepared a paper-based fluorescent biosensor for the determination of Hg^2+^ [74] by immobilizing the cells in the alginate hydrogel, forming a cells-alginate hydrogel encapsulated system which was attached to the paper strip. The developed biosensor was able to detect Hg^2+^ at micro molar concentration in presence of other molecules and ions within 5 min. In addition to these examples, other developed biosensors for the determination of metal ions have been discussed in Table 1 with various information such as the readout system, sensor configuration, response time, detection range, and LOD.

## 4. Biosensors for Detection of Macromolecules

**Figure 3:**
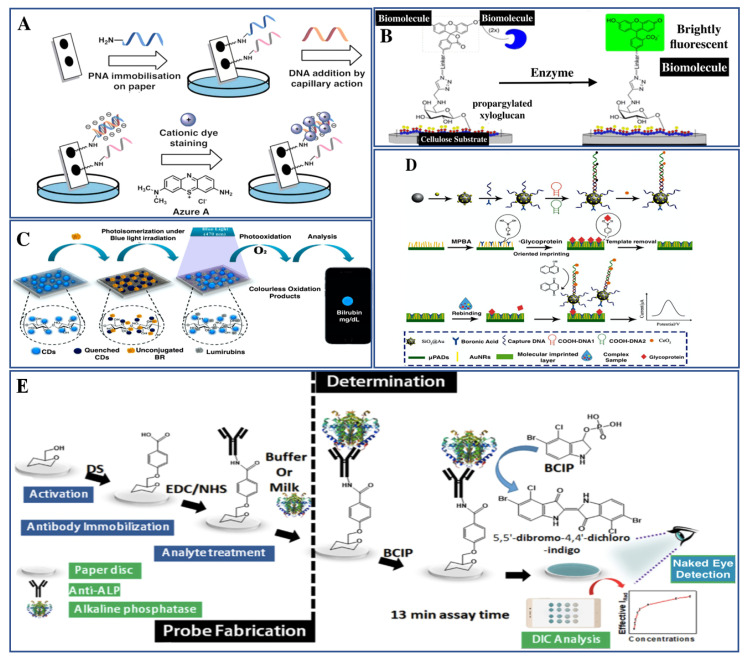
Biosensors for detection of macromolecules. (**A**) Fabrication of DNA sensor by immobilization of acpc PNA (D-prolyl-2-aminocyclopentanecarboxylic acid—peptide nucleic acid) through covalent bonding on the cellulose paper and cationic dye Azure A is used for signal detection (reproduced with permission from [83]). (**B**) Surface tethering of the cellulose-based sensor for the identification of Esterase enzyme by using the flu orogen (reproduced with permission from [90]). (**C**) Illustration of bilirubin sensor using photoluminescent carbon dot sensing probes (reproduced with permission from [88]). (**D**) Fabrication pattern of the paper-based biosensor for determination of glycoprotein (reproduced with permission from [87]). (**E**) Schematic representation of a fabrication and detection method of ALP biosensor (reproduced with permission from [50]).

Macromolecules are relatively high molecular mass polymers composed of thousands of molecules. Macromolecules also show unusual properties that usually do not occur in small molecules. In living organisms, mainly three types of macromolecules are found which help in biological functions such as DNA, RNA, and proteins which are composed of monomer units, and other non-polymer types of macromolecule are also present such as lipid moiety and macrocycles. The developed cellulose-based sensors for the detection of various macromolecules are discussed in this section. A cellulose paper-based sensor has been fabricated for the visual detection of DNA by Jirakittiwut et al., 2015. The biosensor was developed by immobilization of acpc PNA (D-prolyl-2-aminocyclopentane-carboxylic acid PNA) on cellulose paper by divinyl sulfone-mediated conjugation [83]. PNA is an artificial mimic of the DNA that acts as a probe for the sensing of DNA. The interaction between PNA and DNA is based on the difference of charges on these molecules. PNA is a neutral molecule while DNA is a negatively charged molecule and thus provides a unique chance of interaction. The developed biosensor is highly specific and was even able to differentiate between the genes having the single base-pair mutation. The activity of the biosensor is checked using human leukocyte antigen and 26th mutations of thalassemia. The signal detection of the biosensor has been coupled with the cationic dye, Azure A, which lowers the detection limit (Figure 3A). Another paper-based biosensor was designed by Mohanraj et al., 2020, for the investigation of double-stranded DNA (dsDNA). The sensor was fabricated using graphene nanosheets through electrochemical exfoliation of the biomass, derived from corncob [84]. This paper-based graphene sensor can directly detect electrolytes without the requirement of sample preparation. The sensing of dsDNA was based on the oxidation of adenine and guanine within the detection range from 2 × 10^−4^ ng mL^−1^ to 50 × 10^−4^ ng mL^−1^ and the LOD of 0.68 pg mL^−1^. More information about other developed biosensors for nucleotide detection has been tabulated in Table 2.

Cellulose-based sensors are not only for the nucleotides but are also developed for the sensing of different types of proteins. In this direction, several proteins have been detected using a novel sensor system. In one such example, the transcription factor (TF) has been detected, which is a DNA binding protein essential for gene regulation. Lin et al., 2019, have developed a paper-based biosensor for the visual detection of TF, which was fabricated through dopamine coating onto the cellulose paper and was used to obtain the analytical signals [85]. Characterization of the developed sensor was performed by FTIR and other techniques. The developed biosensor was analyzed for the target NF-κB p50, which is based on the Exo III-mediated cycling amplification reaction and the response was investigated by a color change. It was also hypothesized that the proposed biosensor is generic and can be extended towards different biomolecules by changing the recognition system, and thus may provide a low-cost, disposable, portable biosensing device. Another type of protein molecule is alkaline phosphatase (ALP), a metalloprotein that is inherently present in milk and acts as a biomarker for the investigation of pasteurized milk. In this direction, Mahato et al., 2019, developed a paper-based biosensor for the visual identification of ALP integrated with a smartphone system [50]. The sensor was fabricated through immobilization of the ALP antibody on the top of the paper. The ALP was detected through an immune complexation reaction between probe and ALP that forms a blue-green precipitate in the presence of 5-bromo-4-chloro 3-indolyl phosphate (Figure 3E). The quantification was performed using the digital image colorimetry technique and the detection range was observed between 10 to 1000 U/mL; LOD was found to be 0.87 (±0.07) U/mL. This study helps in developing an affordable biosensor for checking the quality of milk in miniaturized and personalized settings.

Apart from these, cellulose-based devices have also been explored for the sensing of glycoproteins, which plays a critical role in cell division, cell signaling, cell migration, and also as a biomarker for various disease diagnoses [86]. Another paper-based electrochemical biosensor has been developed for the ultra-sensitive identification of glycoprotein ovalbumin (OVA) by Sun et al., 2019. The biosensor was fabricated through the introduction of Au nanorods on the cellulose paper, which acted as a matrix for the preparation of boron ate-based molecularly imprinted polymers (MIPs) [87]. For biosensor fabrication, AuNPs were embedded on the top of SiO_2_ nanoparticles and the formed SiO_2_@Au were anchored with the dsDNA to enhance the signal. CeO_2_ nanoparticles were used as an indicator that binds with the dsDNA, which leads to the formation of the SiO_2_@Au/dsDNA/CeO_2_ signal tag. The boron ate affinity-based MIPs were immobilized on a paper matrix to recognize the target glycoprotein OVA, through the covalent bonding formation between the boronic acid and glycoprotein. The detection range of the developed biosensor was measured to be 0.001 ng/mL to 1μg/mL and the LOD was 0.87 pg/mL (S/N = 3). Cellulose-based sensors have also been fabricated for the identification of another protein such as Bilirubin, which is related to jaundice and other clinical conditions. Tabatabaee et al., 2019, have prepared a photo lumines cent nano paper-based biosensor for early diagnosis of jaundice through the detection of Bilirubin in infants’ blood samples. This provides an easy, effective, non-toxic, disposable, and inexpensive biosensor with a smartphone readout [88]. Smartphone readout systems provide great potential for point-of-care and point-of-need platforms [89]. It was fabricated by embedding the photoluminescent carbon dot sensing probes in BC nano paper substrate. Quenching of the photoluminescence was observed in the presence of Bilirubin, which acts as a quencher and was selectively recovered upon blue light (λ = 470 nm) exposure. The resulting intensity of the biosensor was found to be linearly proportional to the amount of Bilirubin present in the sample with a detection range of 2 to 20 mg dL^−1^.

Furthermore, the cellulose-based matrix is explored for fabricating the biosensor for the detection of numerous types of enzymes. A cellulose-based calorimetric biosensor was manufactured by Ling et al., 2019, for the identification of the enzyme human neutrophil elastase (HNE) [91]. HNE is a serine protease secreted from neutrophil at the time of chronic wounds and leads to the breakdown of proteins responsible for healing. The biosensor was developed through immobilization of HNE peptide to the cotton and wood nanocellulose. Cotton CNCs show a higher degree of sensing than wood cellulose nanofibrils. The sensitivity of cotton CNCs colorimetric sensor was observed to be less than 0.005 U/mL. Apart from the HNE, cellulose-based sensors also developed for other enzymes such as acetylcholinesterase (AChE), which plays a significant role in the hydrolysis of the neurotransmitter acetylcholine into choline and acetic acid. AChE is present at the neuromuscular junction and in the chemical synapse, which terminates the synaptic transmission [92]. A cellulose nanofiber-based biosensor has been manufactured for the identification of AChE by Wang et al., 2021, by grafting the DNA aptamer onto CNF. For the detection of AChE activity, CNF-DNA was combined with silver to develop CNF-DNA-AgNCs and chemical characterization was performed by FTIR, XPS, scanning electron microscopy (SEM), and TEM analysis [93]. Acetylthiocholine (ATCh) has been used as a substrate for AChE, causing hydrolysis of ATCh, converting it to thiocholine that reacted with the CNF-DNA-AgNCs. This reaction overall generated the analytical signals that were calibrated to detect the target compound. The sensitivity of the developed biosensor for AChE concentration was observed to be 0.053 mU/mL. Both these and the cellulosic matrix were also designed for the sensing of various macromolecules, as described in Table 2 with their design parameters and analytical performances.

## 5. Biosensors for Detection of Cells

**Figure 4:**
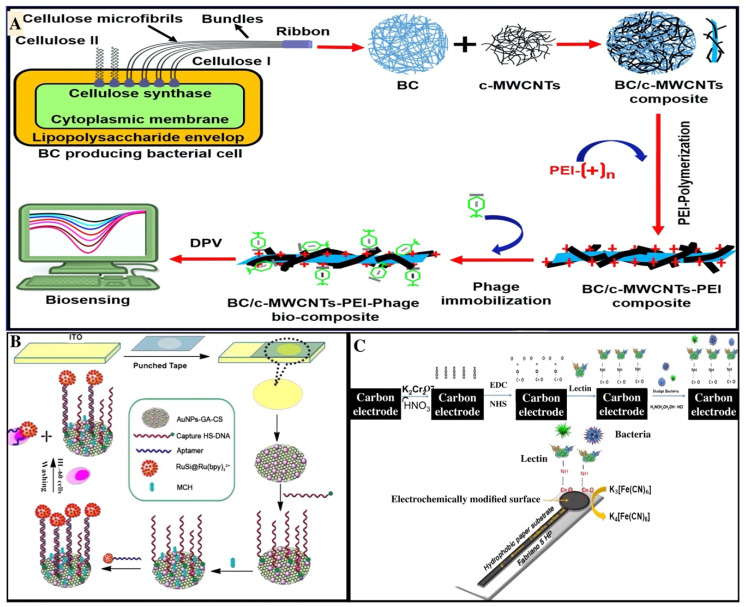
Biosensors for detection of cells. (**A**) Fabrication scheme of the sensor for identification of *S. aureus* cells using (**B**,**C**) matrix (reproduced with permission from [101]). (**B**) Pictorial depiction of paper-based ECL biosensor for the sensing of HL-60 cancer cells (reproduced with permission from [116]). (**C**) Representation of steps involved in electrode surface modification and the bacterial detection by the SPCE (Reproduced with permission from [117]).

In addition to small molecules, macromolecules, and metal ions detection cellulose-based biosensors are also being developed for the visualization of cells. These biosensors are effective in determining both prokaryotic and eukaryotic cells. Here, we have discussed the previously developed sensors, focusing upon the identification of various kinds of bacteria, viruses, fungi, and also some cancer cells. In recent years, cellulose-based biosensors have been developed for bacteria detection where some interesting work has been carried out towards *Staphylococcus aureus* which produces enterotoxins and is the main reason for food poisoning and skin infections [99,100]. For the safety of food and to prevent humans from bacterial infection, it is required to focus on quick responsive detection of *S. aureus.* An electrochemical cellulose-based biosensor was manufactured for the early determination of living *S. aureus cells* from a sample of food containing both live and dead cells by Farooq et al., 2020. The biosensor was fabricated by immobilizing the bacteriophage onto a modified surface of the BC matrix. BC has a poriferous and fibrous structure that provides a larger effective surface for the impregnation of carboxy late multiwalled carbon nanotubes (c-MWCNTs), which enables greater density and phage immobilization. BC/c-MWCNTs nanocomposites modified through surface polymerization with polyethylene eimine provide a positive charge, which assists in the correct orientation of the phage on the matrix. Detection of anti-staphylococcus activity of the immobilized phage was measured through optical density and the density was determined through confocal microscopy. The developed biosensor effectively detects *S. aureus* up to 3 and 5 CFU⋅mL^−1^ in the phosphate buffer saline and milk sample, respectively, using DPV in 30 min [101]. The biosensor shows high sensitivity, specificity, accuracy, and stability up to six weeks at 4 °C (Figure 4A). The investigation of bacterial pathogens is a main problem for the food industry and also for public health. *Escherichia coli*, *Staphylococcus aureus*, and *Listeria monocytogenes* are the three most crucial microorganisms which disperse through food and cause food borne diseases [102,103]. Another paper-based biosensor was fabricated for the identification of pathogenic bacteria by Liu et al., 2015. The biosensor relied on multiplex asymmetric PCR and a paper-based matrix. The probe was conjugated with AuNPs for the visual sensing of genomic DNA of the pathogenic microorganism. The detection limit of the developed method was found to be 1 pg/μL genomic DNA in standard conditions [103]. Additionally, another paper-based sensor was developed for the detection of a highly pathogenic microorganism: *Escherichia coli* O157:H7 present in food samples through naked eye detection by You et al., 2021. The biosensor was fabricated by using Poly-L-lysine-coated starch magnetic particles (PLL@SMPs) for magnetic separation. The signal amplification is performed by using an antibody conjugated with Horseradish peroxidase and 3, 3′, 5, 5′-tetramethylbenzidine. The developed PLL@SMPs show an efficiency of >90% in a large volume for the target bacteria. The LOD of the biosensor was observed to be as low as 30.8 CFU/mL with a probability of 95% [104]. This technique may offer an easy, sensitive, and specific process for the evaluation of the food and surrounding samples. Apart from the bacteria, interestingly, cellulose-based sensing devices have also been used for the detection of viruses. Pseudorabies virus (PRV) causes porcine pseudorabies, which is an acute virulent that is infectious to livestock and wildlife. Due to the swine infection with PRV, there is a great financial loss for the pig industry which requires the urgent diagnosis of PRV. Recently, Huang et al., 2021, have manufactured a paper-based biosensor integrated with a smartphone for the diagnosis of wild-type PRV infection [105]. For detection, latex beads have been used for labeling PRV and the coated test line with PRV gE-m Abs. The signals were recorded and processed using the smartphone’s light detector. The developed biosensor indicates excellent sensitivity, selectivity, and rapid detection of PRV within 15 min.

Nowadays, cytosensing is also extensively used for the detection of multiple types of cancer cells [106,107]. There are various surface biomarkers such as MUC-1 (Mucin 1), carcino embryonic antigen (CEA), BRCA1, BRCA2, HERS, folic acid receptors, sialic-acid associated glycoprotein, and many other protein/glycoprotein biomarkers [108] that are recognized by aptamers [109], antibodies, and nano-enzymes immobilized on cellulose matrix [110]. More emphasis has been given on whole cell-system-based biosensing and a microfluidic system for targeting different antigens expressed by cancer cells at the onset of the disease. Cellulose-based multiplexed biosensor systems and microfluidic paper-based biosensing devices are utilized nowadays for detecting multiple surface antigen-specific to cancer synchronously. Kumar et al. (2016) portrayed electrochemical biosensors for the investigation of CEA cancer biomarker [111]. The sensor was developed using a novel strategy comprising systematic layering of the components. The performance of the sensor was comparable and shows the CEA in clinical levels. Liang et al. (2016) detected the cancer cells by multiplexed sensing panel, which is based on a microfluidic paper-based biosensor [112]. The author uses mesoporous silica nanoparticles that were encapsulated with cadmium telluride QDs and DNA aptamers were immobilized onto it, offer ing great sensitivity, specificity, and efficiency against MCF-7, HL-60, K562 cancerous cells using the quenching ability of GO [112]. When tumor cells were injected on the microfluidic paper analytical device, the fluorescence intensity of QDs increases [113]. This kind of microfluidics paper analytical device could be utilized for the identification of metastatic phases of cancerous cells. In another study, Shi, Ma et al., 2019, used an SPCE for the sensing of cancer antigen 125, which was fabricated on a pure cellulosic paper [114]. Dickert et al. created an ABO blood grouping-based biosensor system. In this device, they fabricated the polyurethane imprints on which different surface antigens were identified based on the type of blood group [115]. In an interesting study by Feng, Liu, et al., 2014, a paper-based electrochemiluminescence (ECL) biosensor has been developed for the detection of HL-60 cancer cells. The biosensor was fabricated by coating the AuNPs and graphene on the surface of porous filter paper in a systematic manner. The aptamer used was flagged with silica nanoparticles conjugated with Ru(bpy)_3_^2^^+^ and showed a high affinity towards the target cell (Figure 4B). ECL intensity directly shows the quantity of the HL-60 cancer cells with the detection range of 56 to 56 × 10^5^ cells/mL [116].

Severe acute respiratory syndrome coronavirus 2 (SARS-CoV-2) outbreaks were announced as a global pandemic by WHO. Rapid and sensitive detection of the virus has become the need of the hour and various methods have been explored by scientists across the globe [118,119,120,121,122]. In recent research, a paper-based electrochemical biosensor has been fabricated for the investigation of SARS-CoV-2 [123]. A label-free paper-based biosensor was manufactured by Yakoh et al., 2021, targeting the SARS-CoV-2 antibodies even excluding the need for a specific antibody. The appearance of SARS-CoV-2 antibodies would obstruct the redox reaction of the redox indicator, and the response was measured as a decrease in the current. The recorded result was proven effective when compared with clinical reports, showing satisfactory results [123]. This example clearly suggests the importance of cellulose matrix even in the design and development of biosensors for pandemics. Further examples of cellulosic sensors for the sensing of cells are mentioned in Table 3 with their analytical details.

## 6. Conclusions and Future Prospects

Cellulose is a widely used material showing various advantages over other materials. Cellulose exhibits unique properties such as biocompatibility, flexibility, mechanical strength, biodegradability, electrical properties, and cost-effectiveness. Apart from the cellulose derived from plants, bacterial cellulose, which is obtained from bacteria, shows more advantageous properties such as mechanical strength, water-holding capacity, and biocompatibility. These advantages of cellulose have made it a significant matrix substance in recent times for the fabrication of biosensors. Many applications have been reported concerning cellulose-based sensing of biomarkers for various disease conditions such as cancer, diabetes, liver and kidney disorders, etc., and also in the detection and monitoring of food quality and various pollutants and heavy metals present in surrounding samples, such as in wastewater. Owing to the significant advances, various methods have been examined to modify cellulose to nanocomposite material, which shows unique structures and desirable properties. In this review, a detailed discussion has been carried out on various modifications required for the formation of cellulose matrix and engineering aspects of the cellulose-based sensors for their applications in different areas.

These sensors are used for sensing the numerous types of small molecules, macromolecules, metal ions, and cells, which are discussed through the numerous examples of sensors. We have tried to include the fabricated sensors to date for various categories of molecules possible, and the techniques/methods used for their modifications. Despite the great progress achieved, there are still some issues that need to be addressed. Recent advances in cellulose-based biosensors typically exhibiting a single function type only and lack the multi-functionality, which leads to its limitation in function and also affects the performances and customer satisfaction. In the future, numerous innovative technologies could be introduced in developing cost-effective multifunctional cellulose-based biosensors.

## Figures and Tables

**Table 1:** List of developed biosensors using cellulose matrix material for the detection of small molecules and metal ions. (NR: not reported).

Sr. No	Analyte	Detection	Sensor Configuration	Response Time	Real Sample	Detection Range	Limit of Detection	Reference
1	Glucose	Amperometric, CV	GOx immobilized on cellulose paper	NR	Soda beverages	1 to 5 mM	0.18 mM	[21]
2	Glucose	Conductometric	GOx immobilized on nanocomposite of cellulose–tin oxide	NR	NR	0.5 to 12 mM	NR	[54]
3	Glucose	Colorimetric	Sulphated and non-sulfated cellulose nanocrystal/magnetite film	NR	Sweat and saliva	low as 5 mM	NR	[55]
4	Glucose	Voltammetric	Polypyrrole-cellulose nanocrystal-based composites with GOx	NR	NR	1.0 to 20 mM	(50 ± 10) μM	[68]
5	Glucose	Cyclic Voltammetry (CV)	Ag@ SiO_2_-PEG metalloid polymer nanoparticle functionalized with graphene oxide	NR	Urine, Serum	0.1 to 20 mM	NR	[75]
6	Phenol	Voltammetric	Nanocrystalline cellulose CdS QDs tyrosine-based biosensor	NR	Water	5 to 40 μM	0.082 μM	[58]
7	Triethylamine	Fluorescence	Cellulose-based Phen-MDI-CA sensor	NR	NR	NR	0.90 μM	[60]
8	Ethylenediamine	Fluorescence	Cellulose-based Phen-MDI-CA sensor	NR	NR	NR	0.99 μM	[60]
9	Fluazinam	Fluorescence	Disulfide quantum dots (MoS_2_ QDs) cross-linked into cellulose membrane	NR	Food	10 to 800 μM	2.26 μM	[62]
10	Aflatoxin B1	Amperometric	Carbon nanofibers derived from BC and coupled with AuNPs	NR	Wheat	0.05 to 25 ng mL^−1^	0.027 ngmL^−1^	[63]
11	Phenylalanine (Phe)	Colorimetric	Paper-based detection of Phe using phenylalanine ammonia-lyase hybrid nanoflowers	10 min	Urine	60 to 2400 μM	NR	[67]
12	H_2_O_2_	Amperometric	Gold nanoparticles–bacterial cellulose nanofibers (Au–BC)-based sensor	1 s	Disinfector	0.3 μM to 1.00 mM	0.1 μM	[76]
13	Ethanol	Amperometric	Paper-based sensor modified with CB/PBNPs nanocomposite	NR	Beer	up to 10 mM	0.52 mM	[77]
14	Atrazine	Optical	Paper-based algal sensor for nanoencapsulated atrazine	NR	Water	0.5 to 200 nM	4 pM	[78]
15	Cu^2+^	Colorimetric	Liquid cellulose biosensor using a facile one-pot process	10 s	Urine and serum	NR	1.9309 and 1.9154 ppm	[69]
16	Ag^+^	Colorimetric	Cellulose modified to DAC-Tu biosensor	10 min	Water	NR	10^−6^ mol/L	[70]
17	Zn^2+^	SPR optical	Gold thin film modified with a nanocrystalline cellulose	NR	NR	low as 0.01 ppm	NR	[73]
18	Hg^2+^	Fluorescent	Cells-alginate hydrogel paper-based sensor	5 min	Wastewater	NR	NR	[74]
19	Mn^7+^	Fluorescence	Nitrogen, aluminium co-doped cellulose-based carbon dots (N/Al-CDs)	NR	Water	0 to 100 μM	46.8 nM	[79]
20	Fe^3+^	Fluorescence	Nitrogen-doped carbon dots anchored on BC	10 min	NR	0.5 to 600 μM	84 nM	[80]
21	Carbonfuran	Colorimetric	Whatman paper used in ULOC device	3 min	Apple	0.01–5.00 mg L^−1^	0.05 mg Kg^−1^	[81]
22	Ochratoxin A	CV, EIS	Cellulose nanofibrous matrix labelled with aptamer probe	NR	Coffee	0.002–2 ng mL^−1^	0.81 pg mL^−1^	[82]

**Table 2:** List of developed biosensors using cellulose matrix material for the detection of macromolecules. (NR: not reported).

Sr. No	Analyte	Detection	Sensor Configuration	Response Time	Real Sample	Detection Range	Limit of Detection	Reference
1	DNA	Colorimetric	Acpc PNA on cellulose paper by DVS conjugation	NR	Human leukocyte antigen alleles	low as 200 nm	NR	[83]
2	dsDNA	CV, EIS	Paper-based modified electrode sensor	NR	NR	0.2 pg/mL to 5 pg/mL	680 fg mL^−1^	[84]
3	miRNA	Voltammetric	PNA-based paper biosensor	<1 h	Serum	up to 100 nm	6 nm	[94]
4	miR-21	Voltammetric	Cerium dioxide—Au@ glucose oxidase paper-based sensor	NR	Serum	0.001 pm to 1 pm	0.434 fm	[95]
5	ALP	Colorimetric	Paper-based naked eye detection	NR	Milk	10 to 1000 U/mL	0.87 (±0.07) U/mL	[50]
6	Esterase	Fluorescence	Chemoenzymatic method used for modification of cellulose matrix	NR	NR	NR	NR	[90]
7	Transcription factor	Colorimetric	Dopamine coated on the surface of cellulose paper	20 s	NF-κB p50 in biological fluids	NR	NR	[85]
8	Glycoprotein	Voltammetric	Paper-based biosensor for glycoprotein based on boronate affinity tag	NR	Ovalbumin	0.001 ng/mL to 1 μg/mL	870 fg/mL	[87]
9	Bilirubin	Photoluminescence	BC nanopaper-based biosensor through embedding of carbon dot sensing probes	NR	Infant’s blood	2 to 20 mg dL^−1^	NR	[88]
10	Human neutrophil elastase (HNE)	Colorimetric	Immobilizing HNE peptide to the cotton and wood nanocellulose	NR	Chronic wound fluid	Less than 0.005 U/mL	NR	[91]
11	Acetylcholinesterase (AChE)	Fluorescence	DNA aptamer immobilized on the surface of cellulose nanofiber	NR	NR	NR	NR	[93]
12	Interleukin-6	Colorimetric	Paper sensor for IL-6 detection in COVID-19 patients	10 min	Respiratory	up to 10^−1^ ng mL^−1^	1 fg mL^−1^	[96]
13	Suppression of Tumorigenicity 2	CV, EIS	Graphite paper-based disposable sensor through modification of fullerene C_60_	NR	Serum	as low as 414 ag mL^−1^	124 ag mL^−1^	[97]
14.	Bovine haptoglobin	Colorimetric	AuNP/MWCNT-anti-Hpnanobioconjugate paper-based sensor	NR	Serum	0.01 to 0.9 mg/mL	28 μg/mL	[98]

**Table 3:** List of developed biosensors using cellulose matrix material for the detection of cells. (NR: not reported).

Sr. No	Analyte	Detection	Sensor Configuration	Response Time	Real Sample	Detection Range	Limit of Detection	Reference
1	*Staphylococcus aureus*	Voltammetric	Immobilization of bacteriophage onto BC	30 min	Milk, PBS	5 CFU mL^−1^, 3 CFU mL^−1^	NR	[101]
2	*Staphylococcus aureus*	Optical	Paper-based biosensor using a primer-based asymmetric PCR	NR	*nuc* gene	low as 1 pg/μL	NR	[103]
3	*Listeria monocytogenes*	Optical	Paper-based biosensor using a primer-based asymmetric PCR	NR	*Hlya* gene	low as 1 pg/μL	NR	[103]
4	*Escherichia coli*	Optical	Paper-based biosensor using a primer-based asymmetric PCR	NR	*rbfE* gene	low as 1 pg/μL	NR	[103]
5	*Escherichia coli*	Colorimetric	PLL@SMPs-based paper sensor	NR	Food	NR	30.8 CFU/mL	[104]
6	Pseudorabies virus	Colorimetric	Latex beads paper-based sensor using PRV gE-mAb	15 min	Pig serum	NR	NR	[105]
7	HL-60 cancer cell	Electrochemiluminescence	Ru(bpy)^2^ 3^+^-conjugated silica nanoparticle-based	NR	NR	56–5.6 × 10^6^ cells/mL	NR	[116]
8	SARS-CoV-2	Voltammetric	Label-free paper-based biosensor	30 min	Serum	1 ng/mL	NR	[123]
9	*Listeria monocytogenes*	Chemiluminescence	Paper-based sensing device with an immobilized DNA probe	NR	*hlyA*gene	0.194 pmol/L to 19.4 × 10^3^ pmol/L	6.3 × 10^−2^ pmol/L	[124]
10	Papillomavirus	Voltammetric	acpcPNA and graphene-polyaniline modified paper-based biosensor	NR	SiHa cells	10 to 200 nM	2.3 nM	[125]

## Data Availability

Not applicable.
